# Plasma proteome analysis in patients with pulmonary arterial hypertension: an observational cohort study

**DOI:** 10.1016/S2213-2600(17)30161-3

**Published:** 2017-09

**Authors:** Christopher J Rhodes, John Wharton, Pavandeep Ghataorhe, Geoffrey Watson, Barbara Girerd, Luke S Howard, J Simon R Gibbs, Robin Condliffe, Charles A Elliot, David G Kiely, Gerald Simonneau, David Montani, Olivier Sitbon, Henning Gall, Ralph T Schermuly, H Ardeschir Ghofrani, Allan Lawrie, Marc Humbert, Martin R Wilkins

**Affiliations:** a Department of Medicine, Imperial College London, Hammersmith Campus, London, UK; b National Heart and Lung Institute, Imperial College London, Hammersmith Campus, London, UK; c National Pulmonary Hypertension Service, Imperial College Healthcare NHS Trust, Hammersmith Hospital, London, UK; d Department of Infection, Immunity and Cardiovascular Disease, University of Sheffield, Sheffield, UK; e Sheffield Pulmonary Vascular Disease Unit, Royal Hallamshire Hospital, Sheffield, UK; f University Paris-Sud, Université Paris-Saclay, Le Kremlin-Bicêtre, Paris, France; g AP-HP, Service de Pneumologie, Hôpital Bicêtre, Le Kremlin-Bicêtre, Paris, France; h Inserm UMR_S 999, Hôpital Marie Lannelongue, Le Plessis-Robinson, Paris, France; i University of Giessen and Marburg Lung Center, Member of the German Center for Lung Research (DZL), Giessen, Germany

## Abstract

**Background:**

Idiopathic and heritable pulmonary arterial hypertension form a rare but molecularly heterogeneous disease group. We aimed to measure and validate differences in plasma concentrations of proteins that are associated with survival in patients with idiopathic or heritable pulmonary arterial hypertension to improve risk stratification.

**Methods:**

In this observational cohort study, we enrolled patients with idiopathic or heritable pulmonary arterial hypertension from London (UK; cohorts 1 and 2), Giessen (Germany; cohort 3), and Paris (France; cohort 4). Blood samples were collected at routine clinical appointment visits, clinical data were collected within 30 days of blood sampling, and biochemical data were collected within 7 days of blood sampling. We used an aptamer-based assay of 1129 plasma proteins, and patient clinical details were concealed to the technicians. We identified a panel of prognostic proteins, confirmed with alternative targeted assays, which we evaluated against the established prognostic risk equation for pulmonary arterial hypertension derived from the REVEAL registry. All-cause mortality was the primary endpoint.

**Findings:**

20 proteins differentiated survivors and non-survivors in 143 consecutive patients with idiopathic or heritable pulmonary arterial hypertension with 2 years' follow-up (cohort 1) and in a further 75 patients with 2·5 years' follow-up (cohort 2). Nine proteins were both prognostic independent of plasma NT-proBNP concentrations and confirmed by targeted assays. The functions of these proteins relate to myocardial stress, inflammation, pulmonary vascular cellular dysfunction and structural dysregulation, iron status, and coagulation. A cutoff-based score using the panel of nine proteins provided prognostic information independent of the REVEAL equation, improving the C statistic from area under the curve 0·83 (for REVEAL risk score, 95% CI 0·77–0·89; p<0·0001) to 0·91 (for panel and REVEAL 0·87–0·96; p<0·0001) and improving reclassification indices without detriment to calibration. Poor survival was preceded by an adverse change in panel score in paired samples from 43 incident patients with pulmonary arterial hypertension in cohort 3 (p=0·0133). The protein panel was validated in 93 patients with idiopathic or heritable pulmonary arterial hypertension in cohort 4, with 4·4 years' follow-up and improved risk estimates, providing complementary information to the clinical risk equation.

**Interpretation:**

A combination of nine circulating proteins identifies patients with pulmonary arterial hypertension with a high risk of mortality, independent of existing clinical assessments, and might have a use in clinical management and the evaluation of new therapies.

**Funding:**

National Institute for Health Research, Wellcome Trust, British Heart Foundation, Assistance Publique-Hôpitaux de Paris, Inserm, Université Paris-Sud, and Agence Nationale de la Recherche.

## Introduction

Idiopathic and heritable pulmonary arterial hypertension constitute a rare disease group characterised by an imbalance in endothelial-derived vasoactive factors, inflammation, and structural remodelling of pulmonary vessels.^1^ The resultant pressure load on the right ventricle causes premature death from heart failure.^1^, ^2^ The incidence of pulmonary arterial hypertension is estimated at 1–7·6 per million per year and cases of idiopathic or heritable pulmonary arterial hypertension account for 0·9–2·6 per million per year.^3^ Estimated 3-year survival is 58–74%,^2^, ^4^ but the disease is heterogeneous; several biological mechanisms^1^ and a range of variants in several genes^5^ have been linked to pathogenesis, and life expectancy is variable. Regular assessment of disease severity and prognosis is necessary to guide clinical management. The existing guidelines recommend a combination of established prognostic parameters on the basis of clinical assessment, imaging, and biochemistry.^6^ These clinical parameters are not always available for each patient visit and existing risk assessments have poor accuracy (with C statistics ranging between 0·57 for the US National Institutes of Health,^7^ 0·59 for the French Registries,^2^ and 0·77 for the REVEAL equation^8^), leaving considerable scope for improvement.^3^

**Research in context**

**Evidence before this study**

We searched PubMed for relevant articles (before March 1, 2017) with search terms including “pulmonary arterial hypertension”, “prognostic”, “proteomics”, and “biomarker”. Several studies report single biomarkers for pulmonary arterial hypertension, usually derived from other diseases, but none have undertaken unbiased screening of large numbers of plasma markers and related these to outcomes in pulmonary arterial hypertension. The prognostication of pulmonary arterial hypertension remains poor. Pulmonary arterial hypertension is diagnosed at cardiac catheterisation. Thereafter a combination of exercise capacity (eg, 6-min walk test), patient-reported symptoms (eg, functional class assessment), echocardiography, and circulating NT-proBNP concentrations—captured in prognostic equations such as the REVEAL score—are used to follow disease progression, response to treatment, and make clinical management decisions. Not all these measurements are made at each visit and some (eg, 6-min walk test) are subject to confounding factors. Better, non-invasive, and objective methods of assessment are needed that can be deployed in the clinical setting.

**Added value of this study**

We did an unbiased screen of 1129 proteins measured in plasma samples collected on routine clinic visits. Measurement of circulating concentrations of nine proteins in combination predicted survival, which outperformed traditional clinical assessments. A prognostic score on the basis of plasma concentrations of the nine proteins was validated in independent cohorts from three countries (UK, France, and Germany) and is relevant to both incident and prevalent cases of pulmonary arterial hypertension. An increase in the panel score over time is associated with increased mortality.

**Implications of all the available evidence**

The guidelines for prognostication in pulmonary arterial hypertension recommend the use of only one blood biomarker, BNP or NT-proBNP, and consideration of a multitude of clinical measures, which when formalised into risk equations perform only moderately well in predicting outcomes. These data suggest that a panel of nine proteins, which report on different pathogenic mechanisms linked to pulmonary arterial hypertension, can be used to stratify patients according to risk and assess response to treatment better than existing clinical tools. Further investigation of the pathways represented in the protein panel might also offer new insights for the development of novel therapies.

The existing management guidelines include the measurement of plasma brain natriuretic peptide (BNP) or N-terminal proBNP (NT-proBNP) concentrations, an indicator of right ventricular function, in the assessment of patients with pulmonary arterial hypertension.^6^ Biomarkers reporting other components of the pathophysiology of idiopathic pulmonary arterial hypertension, such as inflammation (interleukin 6 and growth differentiation factor 15), renal function (creatinine), and iron status (red cell distribution width), also predict clinical outcome,^9^, ^10^, ^11^, ^12^ but none are used routinely. The use of multiple biomarkers could improve risk assessment.

Proteomics offers an unbiased approach to identifying and quantifying multiple biomarkers representative of disease processes. Mass spectrometry-based proteomic analysis of lung tissue, plasma, and cultured cells from patients with pulmonary arterial hypertension has identified a small number of dysregulated proteins.^13^, ^14^, ^15^ Alternative high-throughput strategies exploiting targeted peptide-binding reagents in a multiplex manner permit the screening of large numbers of identifiable proteins. One such technology uses DNA-based aptamer reagents, known as SOMAmers, that are modified to improve binding kinetics.^16^, ^17^

We used a SomaScan array to measure concentrations of 1129 proteins in plasma to identify and validate circulating proteomic signatures that predict survival of patients with idiopathic or heritable pulmonary arterial hypertension.

## Methods

### Study design and participants

In this multicentre, observational cohort study, we identified and analysed four cohorts of patients with idiopathic or heritable pulmonary arterial hypertension from three expert centres recognised internationally as centres of excellence for pulmonary arterial hypertension diagnosis and management in London (UK; cohorts 1 and 2), Giessen (Germany; cohort 3), and Paris (France; cohort 4). The diagnostic criteria for idiopathic or heritable pulmonary arterial hypertension over the course of this study were stable: raised mean pulmonary artery pressure of more than 25 mm Hg, with pulmonary capillary wedge pressure less than 15 mm Hg (and pulmonary vascular resistance [PVR] >3 Wood units) at rest with exclusion of known associated diseases. The guidelines quoted are internationally agreed. Samples from 25 healthy controls were also collected at Hammersmith Hospital for comparison of proteomic and alternative assay measurements.^6^ All samples and data were obtained with informed consent and local research ethics committee approval.

### Procedures

Patients were not fasting and were sampled at their routine clinical appointment visits (397 in total for all patients). Peripheral venous blood samples were collected using EDTA (edetic acid) for cohorts 1, 2, and 3 or sodium citrate Vacutainer tubes (BD Biosciences, Oxford, UK) for cohort 4, immediately put on ice, centrifuged (1300 × g, 15 min) within 30 min of collection, and plasma aliquots were stored at −80°C until required. The plasma samples underwent one freeze–thaw cycle to aliquot 120 μL for the SomaScan assay and provide other aliquots for NT-proBNP and targeted assays. Clinical data were collected within 30 days of blood sampling and biochemical data were collected within 7 days of blood sampling.^11^ We calculated the REVEAL prognostic equation,^8^ and fitted it to the study cohorts. The equation includes categories on the basis of sub-diagnosis, age, sex, renal insufficiency, WHO functional class, systolic blood pressure, heart rate, 6-min walk distance, NT-proBNP, presence of pericardial effusion, percentage predicted diffusing capacity for carbon monoxide, mean right atrial pressure, and PVR. Proteomic analysis was done with SOMAscanV3 (Somalogic Inc, Boulder, CO, USA)^16^ and patient status was concealed to the technicians. The list of 1129 targeted proteins has been reported previously.^17^ To minimise between-experiment variation, bridging samples were included in all experiments; specifically, to check consistency of the measurements we included samples from 24 patients from cohort 1 in all experiments to verify that the measurements from experiment to experiment were comparable. Median variation in relative fluorescence units between experiments was less than 10%, and more than 90% of analytes showed less than 20% variation in average levels between experiments. Following selection of the proteins of interest from analyses of cohort 1 samples, we measured the same proteins again in the same samples used in the proteomic analysis by alternative commercially available assays, each specific for the protein of interest, to check that the two methods agreed; the ELISA and Luminex assays used to validate the SOMAscan measurements are detailed in the appendix (p 3).

### Outcomes

All-cause mortality was the primary endpoint. In a secondary analysis, lung transplantation or death was used as a composite endpoint.

### Statistical analysis

We present differences in protein concentrations by subtraction of log relative fluorescence units. We assessed the association between patient characteristics and biomarkers by Spearman's rank test or Mann–Whitney *U* test and Kruskal–Wallis test for categorical variables.

We did survival analyses using time from sampling to death or censoring. We compared the protein concentrations of survivors and non-survivors (overall survival in cohort 1 and at 2·5 years' follow-up in cohort 2) with Mann–Whitney tests to maximise the power of the protein validation analysis. We used random sample analysis to assess robustness of differences between survivors and non-survivors: we repeated Mann–Whitney analyses 18 times in both discovery and validation (cohorts 1 and 2), each time removing one patient out of six patients in three randomised blocks, with each sample left out of three analyses.

We tested two panel scoring systems. One system was based on a simple count of proteins indicating risk on the basis of receiver operating characteristic (ROC) cutoffs, and the second was a Cox regression model, in which predicted hazard based on continuous biomarker concentrations was calculated on the basis of fitting to the discovery cohort.

For assessment of discrimination, we used ROC curves to compare prognostic discriminatory power of biomarkers. Kaplan–Meier plots illustrated events (deaths) in relation to biomarker levels and predicted risk in Cox models, assessed by the log-rank test. We fitted the simple panel score and REVEAL equation (an accepted clinical score derived from a variety of clinical parameters)^8^ to Cox models to test the additional value and potential clinical use of the panel. For reclassification, we calculated indices—net reclassification index (NRI) and the relative integrated discrimination improvement (IDI) statistic^18^—with R package PredictABEL^19^ for the addition of the prognostic panel score to the REVEAL equation.^8^ We developed the models in cohorts 1 and 2 combined and validated them in cohort 4. Cohort 3 was used to test the performance of the proteins and panel score longitudinally, before and after initiation of targeted therapy. We assessed calibration of the Cox models by comparing predicted mortality of patients against observed mortality using Harrell's rms package in R. We converted variables to Z-scores (SD around mean) before testing by Cox regression. We did calculations with SPSS version 21.0 and R version 3.0.2.

### Role of the funding source

The funders of the study had no role in the study design, data collection, data analysis, data interpretation, or writing of the report. The corresponding author had full access to all the data in the study and had the final responsibility for the decision to submit for publication.

## Results

**Figure 1:**
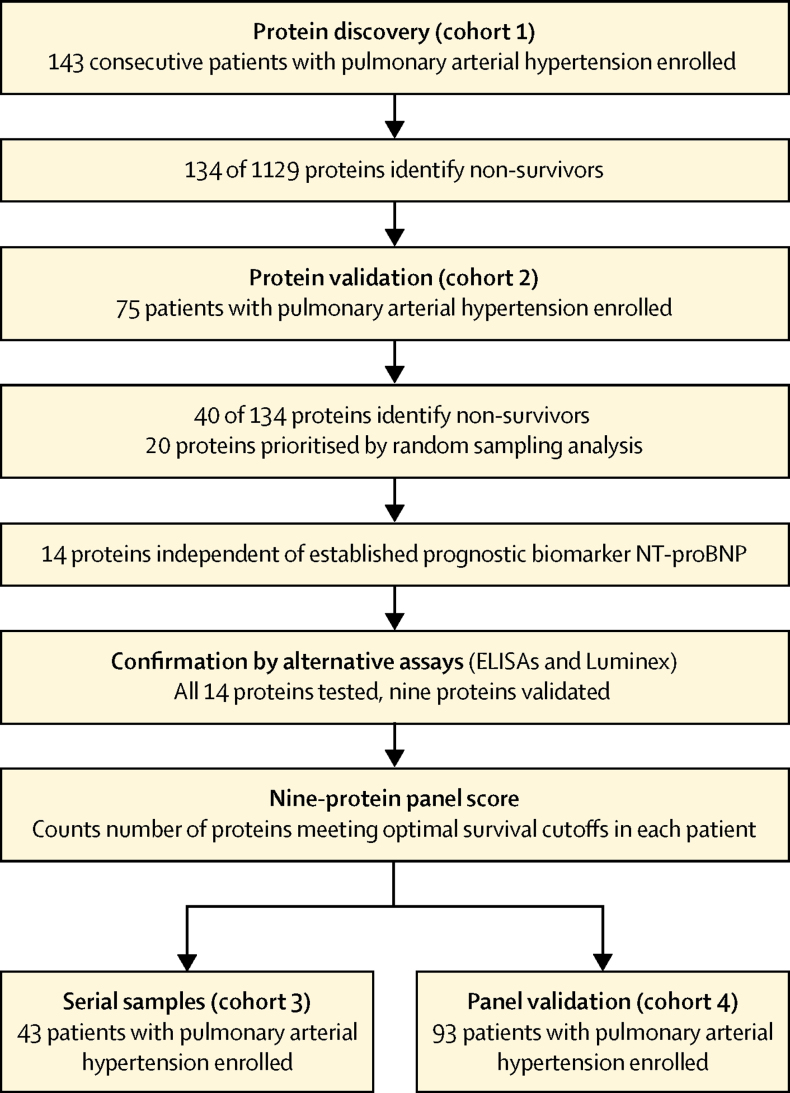
Study design

Cohorts 1 and 2 were censored on May 15, 2014, cohort 3 on May 21, 2015, and cohort 4 on June 1, 2014. We assessed patients for eligibility between Oct 25, 2011, and Aug 13, 2013, for cohort 1, between Oct 24, 2002, and June 22, 2011, for cohort 2, between Aug 27, 2003, and Nov 19, 2012, for cohort 3, and between June 2, 2003, and Dec 23, 2011, for cohort 4 (table 1, figure 1). At the end of the follow-up periods, 18 patients died in cohort 1, 37 patients died in cohort 2, 17 patients died in cohort 3, and 39 patients died in cohort 4; no patients were lost to follow-up. Nine patients (n=3 cohort 1 and n=6 cohort 2) underwent lung or heart and lung transplantation; seven of nine patients who had undergone transplantation died during follow-up.

**Table 1:** Baseline characteristics

		**Cohort 1 (n=143)**	**Cohort 2 (n=75)**	**Cohort 3 (n=43)**	**Cohort 4 (n=93)**
Recruitment period		2011–13	2002–11	2004–11	2003–11
Age, years		53 (41–69)	55 (39–70)	50 (29–61)	54 (39–66)
Sex					
	Females	100 (70%)	45 (60%)	28 (65%)	58 (62%)
	Males	43 (30%)	30 (40%)	15 (35%)	35 (38%)
Ethnic origin					
	White	117 (82%)	59 (79%)	43 (100%)	78 (84%)
	Asian	14 (10%)	11 (15%)	0	5 (5%)
	Black	4 (3%)	3 (4%)	0	8 (9%)
	Other ethnicity or not stated	8 (6%)	2 (3%)	0	2 (2%)
Idiopathic pulmonary arterial hypertension		140 (98%)	71 (95%)	43 (100%)	77 (83%)
Heritable pulmonary arterial hypertension		3 (2%)	4 (5%)	0	16 (17%)
WHO FC					
	Class I	6 (4%)	1 (1%)	0	4 (4%)
	Class II	32 (22%)	14 (19%)	6 (14%)	28 (30%)
	Class III	91 (64%)	41 (55%)	28 (65%)	55 (59%)
	Class IV	14 (10%)	19 (25%)	9 (21%)	6 (6%)
6-min walk, m		339 (144–432)	258 (120–369)	359 (251–425)	390 (300–433)
mPAP, mm Hg		52 (43–62)	51 (46–62)	50 (45–58)	51 (44–61)
mRAP, mm Hg		10 (6–13)	12 (8–17·5)	7 (3–10)	6 (3·5–10)
PAWP, mm Hg		10 (8–14)	10 (7–13)	8 (5–9)	8 (6–10)
CI, L/min/kg/m^2^		2·13 (1·71–2·65)	2·2 (1·71–2·59)	2·23 (1·89–2·60)	2·54 (2·06–3·40)
CO, L/min		4·16 (3·18–5·39)	4·13 (3·00–5·20)	3·80 (3·23–4·41)	4·30 (3·47–5·50)
PVR, Wood units		10·0 (6·0–14·5)	9·3 (7·5–13·1)	11·4 (8·4–15·0)	9·9 (6·4–14·3)
Treatment naive		13 (9%)	19 (25%)	43 (100%)	24 (26%)
Monotherapy					
	CCB	5 (3%)	0	0	1 (1%)
	PDE5	32 (22%)	14 (19%)	0	7 (8%)
	ERA	14 (10%)	16 (21%)	0	26 (28%)
	Prost	1 (1%)	4 (5%)	0	2 (2%)
Dual therapy					
	ERA and PDE5	53 (37%)	10 (13%)	0	21 (23%)
	Prost and ERA	2 (1%)	5 (7%)	0	2 (2%)
	Prost and PDE5	7 (5%)	5 (7%)	0	3 (3%)
Triple therapy		16 (11%)	2 (3%)	0	7 (8%)
Estimated survival					
	1-year follow-up	96%	89%	98%	91%
	2-year follow-up	88%	63%	88%	88%
	3-year follow-up	0	45%	86%	77%
Time after diagnosis sampled, years		3·16 (0·54–7·3)	1·11 (0·37–2·45)	0·41 (0·32–0·89)*	0·88 (0·15–1·82)
Follow-up, years		2·0 (1·6–2·2)	2·5 (1·5–4·9)	6·5 (4·3–9·9)	4·4 (3·0–5·7)

Data are median (IQR), n (%), or n. WHO FC=WHO/New York Heart Association Functional Classification. Shuttle walk=incremental shuttle walk test. mPAP=mean pulmonary artery pressure. mRAP=mean right atrial pressure. PAWP=pulmonary artery wedge pressure. CI=cardiac index. CO=cardiac output. PVR=pulmonary vascular resistance. CCB=calcium channel blocker. ERA=endothelin receptor antagonist. PDE5=phosphodiesterase 5 inhibitors. Prost=prostanoid analogues. IQR=interquartile range.

* Years after diagnosis sampled for second sample shown; baseline samples were taken at diagnosis.

**Figure 2:**
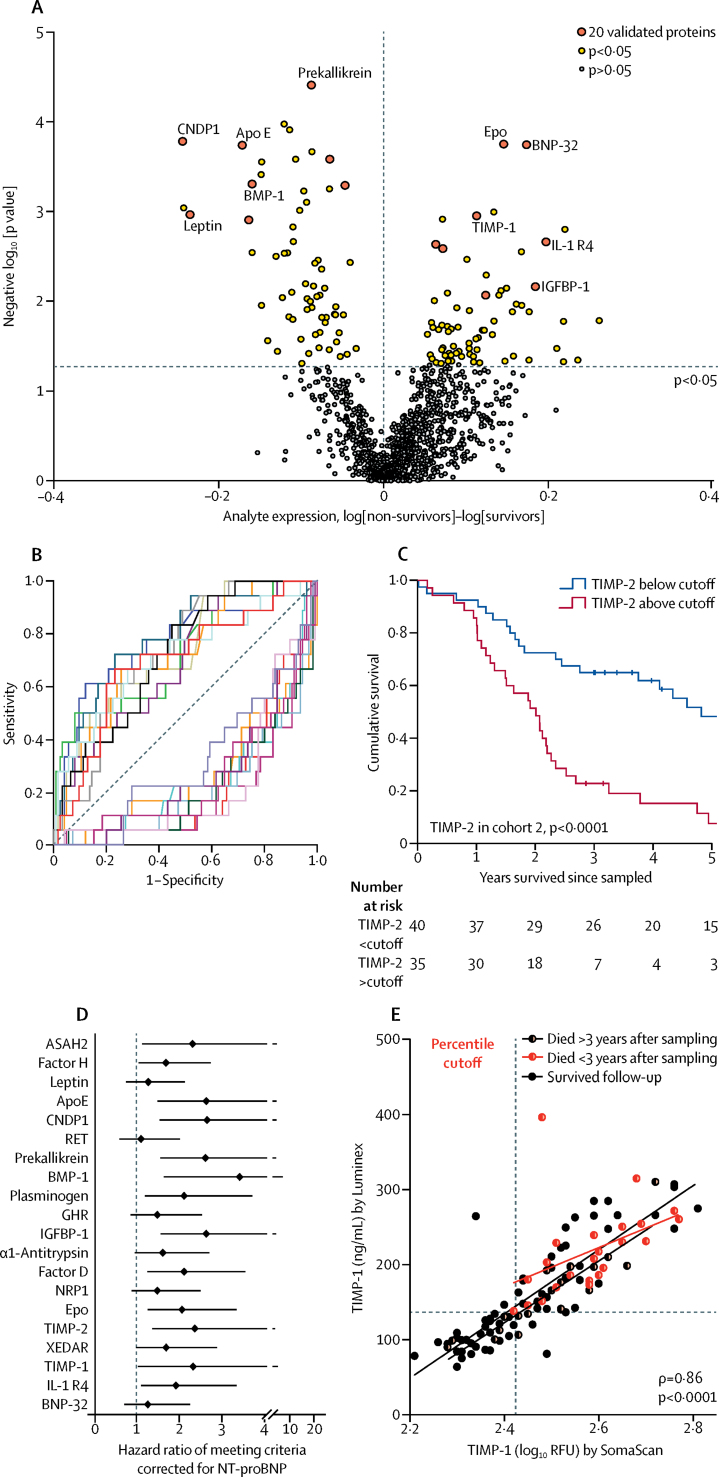
Prognostic protein panel analysis (A) Volcano plot illustrating differences in protein expression between survivors and non-survivors. (B) ROC analysis of 20 selected proteins showing sensitivity and 1 – specificity at cutoffs. (C) Kaplan–Meier survival analysis of patients with idiopathic pulmonary arterial hypertension in cohort 2 divided by TIMP-2 cutoff derived from ROC analysis of cohort 1. (D) Hazard ratios and 95% CI from Cox regression analysis comparing 20 prognostic proteins with established prognostic marker, NT-proBNP. (E) Commercially available ELISA or Luminex assays targeting the 14 independently prognostic proteins used to validate SomaScan measurements in a subset of 80 plasma samples selected from cohort 1 (n=55) and healthy controls (n=25), with samples with high and low concentrations of the analytes chosen. Nine proteins were validated and further studied in cohort 3 (serial samples) and cohort 4 (validation cohort). This scatter-plot illustrates TIMP-1 measurements by SomaScan and Luminex assays in idiopathic pulmonary arterial hypertension cohort 4. Cutoffs for SomaScan and Luminex values derived by percentile equalling ROC-derived cutoff in cohort 1 are indicated by dashed lines. Statistics indicate Spearman's rank correlation. ROC=receiver operating characteristic. RFU=relative fluorescence unit.

Concentrations of 134 proteins were associated with overall survival in cohort 1 (figure 2A). 40 of these proteins were validated as able to differentiate between survivors and non-survivors in cohort 2. 20 prognostic proteins, including BNP, were prioritised by random sampling analysis as the most robust (appendix p 4). These proteins had good specificity and sensitivity in ROC analysis (figure 2B and appendix p 5), and protein concentrations that distinguished between survivors and non-survivors in cohort 1 and performed well in cohort 2 were identified (figure 2C). To ensure the small number of patients with heritable pulmonary arterial hypertension were not confounding, we did an analysis excluding these seven patients and found that the 20 proteins were again significant in both discovery (cohort 1) and validation (cohort 2) analyses (appendix p 6).

We investigated whether the 20 prognostic proteins offered an improvement in risk estimation in addition to the only prognostic protein biomarker currently in use in pulmonary arterial hypertension, namely NT-proBNP. 14 of 20 proteins were each prognostic independent of NT-proBNP in Cox models with death as the primary endpoint (all p<0·05; figure 2D). With transplantation or death as a composite endpoint, all 14 proteins remained significant and independent of NT-proBNP (data not shown).

A significant correlation between values in the SomaScan and the independent ELISA or Luminex assay was shown for nine protein measurements (all p<0·05, Spearman's rank [data not shown])—interleukin-1 receptor-like 1 (IL1R1/ST2), tissue inhibitors of metalloproteinases (TIMP-1 and TIMP-2), plasminogen, apolipoprotein-E (ApoE), erythropoietin (EPO), complement factor H and factor D, and insulin-like growth factor binding protein-1 (IGFBP-1). The measurements for these nine proteins allowed us to derive threshold protein concentrations associated with survival (figure 2E, appendix p 7), and we derived threshold concentrations for each protein in cohort 1 and validated these in cohort 2.

**Figure 3:**
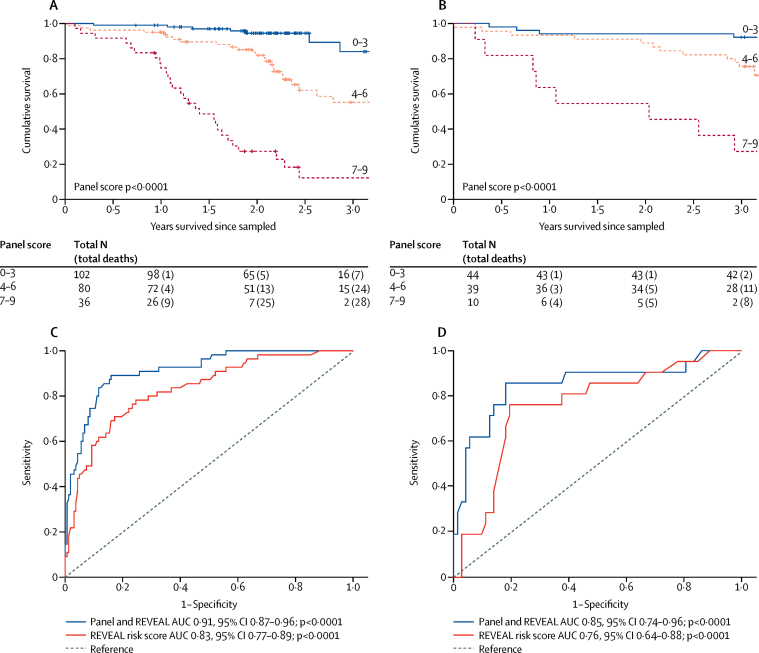
Survival analysis of panel score and established prognostic factors Kaplan–Meier survival estimates in patients with different panel scores, in all patients with idiopathic pulmonary arterial hypertension from (A) cohorts 1 and 2 and (B) cohort 4. ROC analysis of Cox models before and after addition of the prognostic protein panel to the established equation, in all patients with idiopathic pulmonary arterial hypertension from (C) cohorts 1 and 2 and (D) cohort 4. ROC=receiver operating characteristic. AUC=area under the curve.

We used the prognostic thresholds for each of these nine proteins to produce a protein panel score for each patient, whereby each protein indicating risk (ie, when the plasma concentration was above or below the threshold cutoff for survival) added 1 to a patient's score. This calculation produced scores ranging from 0 to 9 for each patient and discriminated non-survivors in discovery (for cohort 1, area under the curve [AUC] 0·93, 95% CI 0·88–0·99) and validation (for cohort 2, 0·86, 0·77–0·94). The simplified scoring of each protein based on a cutoff performed as well as an equation using continuous protein concentrations, which was also derived in cohort 1 and tested in cohort 2 (AUC 0·83, 95% CI 0·75–0·92; appendix p 9). Increasing panel scores clearly distinguished risk groups (figure 3A, appendix p 10).

Removal of any two proteins did not impair the performance of the remaining panel, suggesting no protein was dominant, and emphasising the discriminating power of the combination (appendix p 11). The panel score was also prognostic in a sub-analysis of samples obtained before initiation of therapy, comprising 77 (35%) of 218 patients from cohorts 1 and 2 (appendix p 12) and in groups of patients from cohorts 1 and 2 stratified by age (above and below 50 years; appendix p 13) and bilirubin concentration (21 μmol/L, the upper limit of the normal range in the clinical assay; appendix p 13).

**Figure 4:**
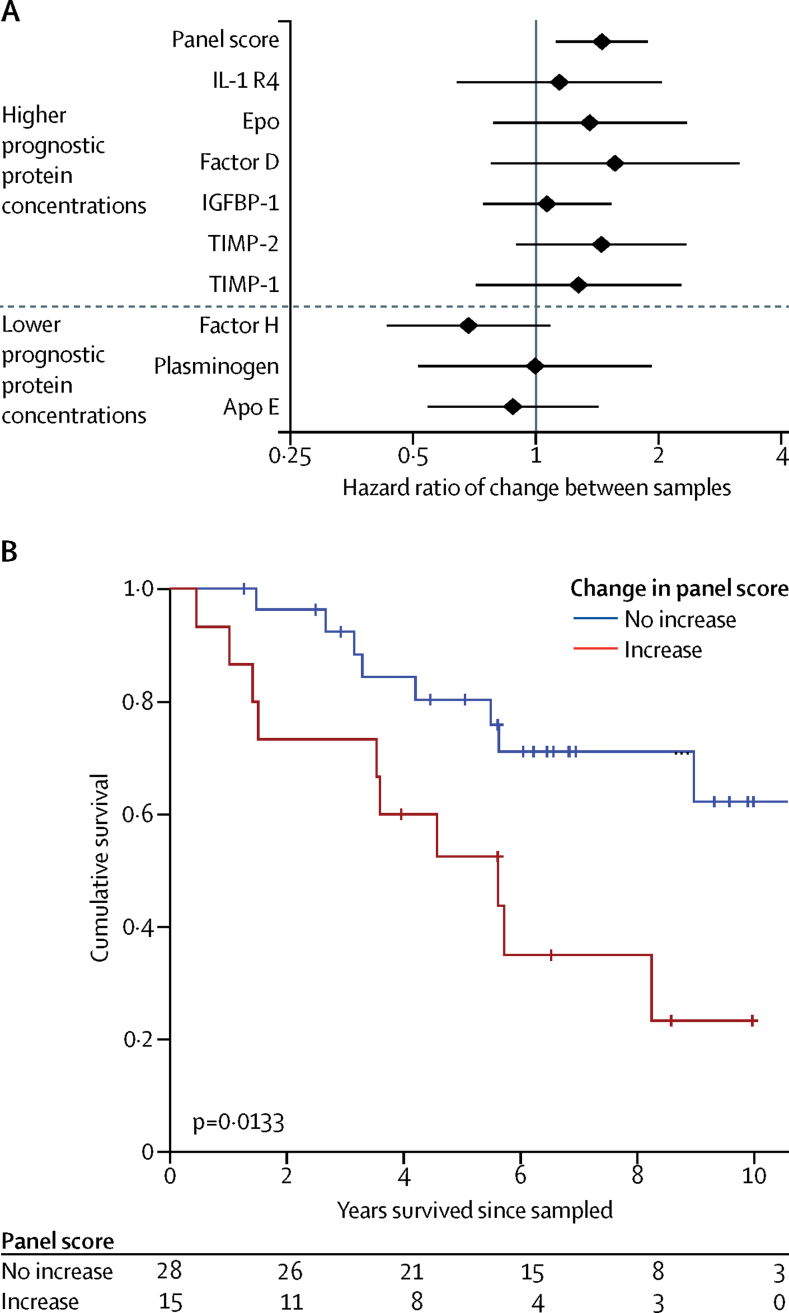
Prognostic value of changes in the protein panel score from diagnosis to after initiation of therapy (A) Cox proportional hazard estimates associated with changes in individual proteins and the overall panel score, showing only the combination of proteins into the score is significantly associated with outcomes. (B) Kaplan–Meier survival estimates in patients with serial panel score measurements (cohort 3), showing an increase in the panel score from diagnosis to after initiation of therapy is associated with poor outcomes.

43 patients were sampled at diagnosis and after initiating therapy in cohort 3 (median time between samples 4 months, IQR 3–10). Although changes in the concentrations of any individual protein, including NT-proBNP, were not associated with outcome, an increasing panel score was prognostic (p=0·0186; figure 4A). Patients whose protein panel score was higher at follow-up than at baseline showed poorer survival than those whose scores remained stable or improved (p=0·0133; figure 4B), which identified patients who had not responded to therapy. These patients had similar clinical characteristics at baseline (appendix p 8). Changes in the panel score appear more sensitive than other measures—eg, the small changes in pulmonary vascular resistance recorded at repeat catheterisation were not associated with survival (appendix p 14).

The protein panel score was further validated in an independent group of 93 patients with idiopathic or heritable pulmonary arterial hypertension from cohort 4 (4·4 years' [IQR 3·0–5·7] follow-up; table 1); an increasing panel score distinguished risk groups (figure 3B, appendix p 10). In panel development (cohorts 1 and 2) and panel validation analyses (in cohort 4), the panel score predicted survival independent of NT-proBNP measurements (appendix p 8).

Results from Cox models confirmed that the REVEAL equation was prognostic and that the protein panel score provided independent prognostic information in both panel development and panel validation (table 2). The categorical NRI indicated that the protein panel score reclassified more patients who died during follow-up at above-average risk and vice versa, while the relative IDI showed a 50–223% relative improvement after addition of the panel score to the model. This outcome means that the protein panel is changing the risk estimates for patients in a significant proportion of individuals, and is providing prognostic information additional to the established equation. In both the panel development and panel validation analyses, the protein panel score improved the C statistic by a similar margin (0·08–0·09; for REVEAL risk score AUC 0·83, 95% CI 0·77–0·89; p<0·0001; for panel and REVEAL 0·91, 0·87–0·96; p<0·0001; figure 3C, D, table 2). Calibration in both model development and validation was similar before and after addition of the panel score (appendix p 15). A combination of the panel score and NT-proBNP as a continuous variable performed very similarly to the combination of the panel score and REVEAL equation in both analyses (appendix p 16).

**Table 2:** Model performance

		**REVEAL equation**	**Panel of nine proteins**
**Performance of equation and panel in combined model**			
C statistic			
	Development	0·83 (0·77–0·89)	0·89 (0·84–0·94)
	Validation	0·72 (0·59–0·84)	0·83 (0·72–0·94)
Hazard ratio in model			
	Development	1·73 (1·36–2·21)	2·44 (1·79–3·33)
	Validation	1·42 (1·01–1·99)	1·9 (1·33–2·72)
**Effect on performance of adding panel to equation**			
Categorical NRI (above/below overall event rate)			
	Development	Reference	0·20 (0·09–0·31)
	Validation	Reference	0·39 (0·07–0·70)
IDI			
	Development	Reference	0·17 (0·09–0·24)
	Validation	Reference	0·13 (0·06–0·20)
Relative IDI			
	Development	Reference	0·50 (0·28–0·72)
	Validation	Reference	2·23 (1·05–3·41)
Δ C statistic			
	Development	Reference	0·083 (0·052–0·114)
	Validation	Reference	0·095 (0·026–0·164)

Hazard ratios from Cox regression analyses of panel score and REVEAL equation, categorical NRI based on overall death—25% (55 deaths in 218 patients) at 2·5 years in development analyses (cohorts 1 and 2) and 23% (21 deaths in 93 patients) at 3 years in validation analyses (cohort 3)—relative IDI (IDI/discrimination slope), and improvement in C statistic (Δ C statistic) after addition of the panel to the REVEAL equation. Model development was performed in cohorts 1 and 2 combined, and validation in cohort 4. NRI=net reclassification index. IDI=integrated discrimination improvement.

## Discussion

To our knowledge, this study is the first to apply high-throughput analysis of the plasma proteome to patients with idiopathic or heritable pulmonary arterial hypertension. The importance of robust statistical interrogation of novel biomarkers versus established criteria in risk prediction models has been emphasised before.^20^ We applied these methods extensively and identified nine proteins that predict survival independent of the established circulating prognostic factor, NT-proBNP. A panel score on the basis of plasma concentrations of these nine proteins, whereby a score increasing from 0 to 9 was associated with increased risk in an individual, improved clinical risk prediction based on NT-proBNP and the REVEAL prognostic equation.^8^ The protein panel was informative when used in incident or prevalent patients, and changes in the panel score after initiating therapy had clinical use by the identification of patients who had not responded to treatment. The protein panel improved model discrimination and reclassification without skewing calibration, and was validated in an independent cohort of patients from a separate expert centre, again independent of established clinical measurements.

BNP was one of the initial 20 prognostic proteins identified following discovery (cohort 1), validation (cohort 2), and random sampling analysis (cohorts 1 and 2), which gave us confidence in our approach. In the final analyses, a panel of nine proteins provided information independent of the most up-to-date risk equation incorporating many clinical variables. The protein components of this panel report on different pathways recognised in the pathophysiology of pulmonary arterial hypertension and collectively are more informative than when used individually. ST2, secreted in response to stretching myocardiocytes,^21^ is a potential biomarker in chronic heart failure,^22^ and circulating concentrations can also reflect inflammation.^23^ Increased TIMP expression and imbalance in matrix metalloproteinase activity is implicated in pulmonary vascular remodelling and disease progression in patients with pulmonary arterial hypertension.^24^ Pulmonary ApoE expression is reduced in patients with idiopathic pulmonary arterial hypertension,^25^ and in experimental models ApoE inhibits the proliferation of pulmonary artery smooth muscle cells and protects against the development of pulmonary arterial hypertension.^26^ The IGF-1 system is known to have a role in vascular pathologies, such as pulmonary arterial hypertension, and IGFBP-1 is one of a family of proteins modulating the effects of IGF-1 on vascular smooth muscle and endothelial cells.^27^ Increased complement factor D expression and loss of the inhibitory factor H predicts dysregulation of the complement system and overactivation of inflammation and innate immunity.^28^ Finally, increased EPO and reduced plasminogen concentrations might reflect the abnormal iron status^29^ and prothrombotic state^30^ of patients with idiopathic pulmonary arterial hypertension. The proteins identified in this study appear to have biological relevance to pulmonary arterial hypertension. None of the proteins identified in this study were the same as those identified, using the same platform, to predict cardiovascular risk in patients with stable coronary heart disease.^17^

Effective clinical management of patients with idiopathic or heritable pulmonary arterial hypertension requires the early recognition of patients who are failing to respond to treatment and need alternative or additional targeted therapies. The existing assessment of patients is dependent on subjective reporting of wellbeing to assign functional class, functional tests such as the 6-min walk test, which lacks specificity, and the availability of data from echocardiography.^6^ The nine-protein panel provides an objective measure that improves the prognostic accuracy of clinical evaluation.^8^ Biomarkers should always be used in clinical context, but it is relevant that the single addition of plasma NT-BNP concentration (a component of the REVEAL equation) as a continuous variable to the panel score performs as well as the panel score plus the REVEAL equation. This observation presents the possibility that the use of the protein panel with NT-BNP might provide a useful point-of-care test for the assessment and early referral of patients with idiopathic or heritable pulmonary arterial hypertension for specialist intervention.

This study represents a comprehensive analysis of the circulating proteome in patients with idiopathic or heritable pulmonary arterial hypertension, leading to the discovery of a panel of proteins capable of predicting mortality more accurately than established measurements. Although the number of proteins assayed represents a broad range of proteins with disparate functions, the proteins studied are limited by the aptamers developed for the assay. Pulmonary arterial hypertension is a rare disease, and the numbers of patients in this study preclude the analysis of interactions between proteins that might improve prognostication. The importance of changes in protein concentrations over time and in response to various therapeutic strategies requires further prospective study, with assessments of the effect of interval between samples. The heterogeneity of pulmonary arterial hypertension and of the patients studied in these cohorts (eg, in terms of age, gender, disease severity, and genetic background) means that the proteins and biological pathways identified in this study might not always be the most important in each individual with pulmonary arterial hypertension. We did the analyses on independent cohorts of patients recruited at three distinct international centres of expertise. The differences in occurrence of death were primarily because of different durations of follow-up and preclusion of long-term survivors in cohort 1 (2011–13) from cohort 2 (2002–11). The effect of different therapies on the concentration of the proteins was not studied and would require sampling of the same subjects before and after initiation of specific therapies at set timepoints. Fasting and dietary status and the method of blood sampling are known to affect proteomic measurements, but we did not study this here. We validated proteomic measurements with alternative experimental methods and similar results were observed in both EDTA-preserved and sodium citrate-preserved plasma. Blood samples were collected alongside routine clinical plasma samples, showing the practical deployment of this protein panel in a clinical setting. The cost of these blood protein measurements would be relatively low compared with more complex clinical procedures. The development of a dedicated assay to measure all nine proteins together would simplify its clinical use.

The prognostic nine-protein panel score powerfully selects subgroups of patients that are likely to have events (death or transplantation), which could be beneficial for targeting aggressive therapeutic strategies or maximising the power of clinical trials. The proteins identified warrant mechanistic evaluation in addition to examination in other distinct disease groups, including other forms of pulmonary hypertension and cardiovascular disease.
