# Supplementary material for: Plasma proteome analysis in patients with pulmonary arterial hypertension: an observational cohort study
Source: Lancet Respir Med. 2017 Sep;5(9):717–26. doi: 10.1016/S2213-2600(17)30161-3 (PMC5573768; doi:10.1016/S2213-2600(17)30161-3)
Supplement: Supplementary appendix [file mmc1.pdf]

THE LANCET
Respiratory Medicine

Supplementary appendix

This appendix formed part of the original submission and has been peer reviewed. We post it as supplied by the authors.

Supplement to: Rhodes CJ, Wharton J, Ghataorhe P, et al. Plasma proteome analysis in patients with pulmonary arterial hypertension: an observational cohort study. *Lancet Respir Med* 2017; published online June 14. http://dx.doi.org/10.1016/S2213-2600(17)30161-3.

Appendix: online supplement

Plasma proteome analysis in idiopathic pulmonary arterial hypertension patients stratified by survival: an observational cohort study

Christopher J. Rhodes[1], John Wharton[1], Pavandeep Ghataorhe[1], Geoffrey Watson[1], Barbara Girerd[6,7,8], Luke S. Howard[2,3], J. Simon R. Gibbs[2,3], Robin Condliffe[5], Charles A. Elliot[5], David G. Kiely[5], Gerald Simonneau[6,7,8], David Montani[6,7,8], Olivier Sitbon[6,7,8], Henning Gall[9], Ralph T. Schermuly[9], H. Ardeschir Ghofrani[9], Allan Lawrie[4], Marc Humbert[6,7,8], Martin R. Wilkins[1]

1: Department of Medicine, Imperial College London, Hammersmith Campus, Du Cane Road, London, W12 0NN, UK

2: National Heart and Lung Institute (NHLI), Imperial College London, Hammersmith Campus, Du Cane Road, London, W12 0NN, UK

3: National Pulmonary Hypertension Service, Imperial College Healthcare Trust NHS, Hammersmith Hospital, Du Cane Road, London, W12 0HS, UK

4: Department of Infection, Immunity & Cardiovascular Disease, University of Sheffield, Sheffield, S10 2RX, UK

5: Sheffield Pulmonary Vascular Disease Unit, Royal Hallamshire Hospital, Sheffield, S10 2JF, UK

6. Univ. Paris-Sud, Université Paris-Saclay, Le Kremlin Bicêtre, France;

7. AP-HP, Service de Pneumologie, Hôpital Bicêtre, Le Kremlin Bicêtre, France;

8. Inserm UMR_S 999, Hôpital Marie Lannelongue, Le Plessis Robinson, France;

9: University of Giessen and Marburg Lung Center (UGMLC), member of the German Center for Lung Research (DZL), Giessen, Germany.

Supplementary Materials

eTable 1 – Methodology for quantification of prognostic proteins in plasma.

eTable 2 – Robustness testing of differences in analytes between survivors and non-survivors in cohorts 1 and 2.

eTable 3 – Performance of prognostic analytes by ROC analysis.

eTable 4 - Sensitivity analysis excluding HPAH patients. Discovery and validation comparisons of protein levels in survivors and non-survivors were performed again excluding 7 HPAH cases. Significance of top 20 robustly prognostic proteins (identified by analysis presented in eTable 2) are shown.

eTable 5 – ROC-derived cut-offs for each of the 9 independent, prognostic proteins validated by alternative assays.

eTable 6 - Clinical characteristics of patients whose protein panel scores did or did not increase after initiation of therapy.

eTable 7 - Cox regression models of panel score against established prognostic marker, NT-proBNP.

eFigure 1 - ROC analysis of 2.5 year survival in cohort 2 predicted by the 9 prognostic proteins, either as a simplified score out of 9 based on cut-offs or using an equation which uses the continuous measurements of each protein for each patient.

eFigure 2 – Mean survival estimates in patients from discovery (cohorts 1 and 2) and validation (cohort 4) divided by panel score.

eFigure 3 – ROC analysis of panel score following removal of any 1 or 2 proteins from the scoring.

eFigure 4 – Sub-analysis of protein panel in patients naïve to PAH targeted therapies.

eFigure 5 - Survival by panel score in PAH patients from cohorts 1 and 2 divided by age and bilirubin levels. A. Patients below 50. B. Patients 50 and above. C. Patients with bilirubin levels below 21 µmol/L and D. above 21 µmol/L.

eFigure 6 – ROC analysis of change in panel score, venous oxygen saturations (VenSO2), NT-proBNP, pulmonary vascular resistance (PVR) and mean pulmonary artery pressure (PAP) from diagnostic catheterisation to follow-up after initiation of targeted therapies.

eFigure 7 - Calibration plots for Cox models.

eFigure 8 - ROC analysis of panel score added to REVEAL equation or NT-proBNP compared to REVEAL equation alone.

Supplementary Methodology: Quantification of prognostic panel proteins in plasma samples

Proteins were quantified in plasma samples using the kits described in eTable 1, following the dilution of samples as described.

Target	Detection	Dilution	Final volume required/kit	Kits required	Sample	Sample Needed	Protocol Makes	Developing time for colour
Epo	ELISA	2	100	RND: DEP00	A	100	125	35
BNP	Clinical assays	10	100	Clinical assays	B	100	125	
Leptin<br>IGFBP-1	Luminex	20	50	RND: custom LXSAHM-02	C	50	300	
IL-1 R4/ST2	ELISA	50	50	RND: DST200	D	50	450	25
TIMP-1<br>TIMP-2	Luminex	400	100	LKTM003 Luminex kit from RND	E	100	300	
Apo E	ELISA	2000	50	RND: DAPE00	F	50	400	60
Factor D	DuoSet	10000	100	RND: DY1824	G	100	350	8
Plasminogen	ELISA	30000	50	Universal Bio EP1200-1	H	150	450	10
Factor H	DuoSet		100	RND: DY4779				20

A - Dilute 100 µl of plasma with 100 µl of reagent diluent from RND to make 200 µl of 2X A

B - Dilute 25 µl of A with 100 µl of diluent to make 125 µl of 10X B

C - Dilute 50 µl of A with 450 µl of diluent to make 500 µl of 20X C

D - Dilute 200 µl of C with 300 µl of diluent to make 500 µl of 50X D

E - Dilute 50 µl of D with 350 µl of diluent to make 400 µl of 400X E

F - Dilute 100 µl of E with 400 µl of diluent to make 500 µl of 2000X F

G - Dilute 100 µl of F with 400 µl of diluent to make 500 µl of 10,000X G

H - Dilute 150 µl of G with 300 µl of diluent to make 450 µl of 30,000X H

Kits for ASAH2 (antibodies-online GmbH, Aachen, Germany, ABIN420312), BMP-1 (ABIN416985), XEDAR (RND/BioTechne, Abingdon, UK, DY1093), Pre-kallikrein (ABIN578408), CNDP1 (ABIN421005) were tested but results did not correlate with the proteomic measurements.

eTable 1 – Methodology for quantification of prognostic proteins in plasma.

Proteins	Differences in median protein expression between survivors and non-survivors				p<0.05 in analyses		
	Cohort 1	Sig.	Cohort 2	Sig.	Cohort 1 /18	Cohort 2 /18	Total /36
**BNP-32**	0.17	0.0002	0.12	0.00002	**18**	**18**	**36**
**IL-1 R4**	0.20	0.0022	0.30	0.000004	**18**	**18**	**36**
**TIMP-1**	0.11	0.0011	0.18	0.00001	**18**	**18**	**36**
**Growth hormone receptor**	-0.16	0.0012	-0.16	0.00003	**18**	**18**	**36**
**Plasminogen**	-0.07	0.0003	-0.07	0.0002	**18**	**18**	**36**
**BMP-1**	-0.16	0.0005	-0.13	0.0002	**18**	**18**	**36**
**Prekallikrein**	-0.09	0.00004	-0.06	0.0022	**18**	**18**	**36**
**RET**	-0.11	0.0001	-0.11	0.0007	**18**	**18**	**36**
**CNDP1**	-0.24	0.0002	-0.17	0.0009	**18**	**18**	**36**
**TIMP-2**	0.07	0.0026	0.10	0.0001	**18**	**18**	**36**
**Leptin**	-0.23	0.0011	-0.21	0.0046	**18**	**18**	**36**
**Factor D**	0.06	0.0023	0.04	0.0038	**18**	**18**	**36**
**Apo E**	-0.17	0.0002	-0.12	0.0066	**18**	**17**	**35**
**NRP1**	0.07	0.0012	0.05	0.0063	**18**	**17**	**35**
**a1-Antitrypsin**	0.10	0.0034	0.09	0.0029	**17**	**18**	**35**
**Epo**	0.15	0.0002	0.11	0.0135	**18**	**16**	**34**
**IGFBP-1**	0.18	0.0069	0.20	0.0027	**16**	**18**	**34**
**XEDAR**	0.12	0.0086	0.18	0.000004	**15**	**18**	**33**
**Factor H**	-0.05	0.0005	-0.03	0.0120	**18**	**15**	**33**
**ASAH2**	-0.15	0.0112	-0.14	0.0030	**16**	**17**	**33**
Factor B	-0.05	0.0142	-0.04	0.0034	13	18	31
PTN	0.07	0.0185	0.14	0.0001	12	18	30
Apo E3	-0.15	0.0003	-0.08	0.0273	18	12	30
IL-2 sRa	0.12	0.0051	0.05	0.0272	17	10	27
PARC	0.11	0.0412	0.16	0.0067	9	18	27
a2-Antiplasmin	-0.04	0.0391	-0.05	0.0028	8	18	26
Kallikrein 7	-0.12	0.0029	-0.07	0.0473	18	8	26
Angiogenin	0.06	0.0173	0.07	0.0089	13	13	26
Afamin	-0.05	0.0225	-0.07	0.0157	9	16	25
C3b	-0.14	0.0276	-0.27	0.0141	9	16	25
ENTP5	-0.09	0.0008	-0.05	0.0416	18	6	24
TFF3	0.12	0.0486	0.12	0.0039	6	18	24
WKFN1	-0.09	0.0094	-0.06	0.0293	14	10	24
Angiopoietin-2	0.10	0.0337	0.16	0.0099	8	16	24
Coagulation Factor V	-0.09	0.0100	-0.09	0.0368	16	8	24
C7	0.07	0.0493	0.11	0.0018	5	18	23
Properdin	-0.06	0.0285	-0.09	0.0171	10	11	21
IL-22BP	-0.11	0.0160	-0.11	0.0289	12	8	20
PCI	-0.05	0.0415	-0.07	0.0499	7	7	14
CDON	-0.07	0.0347	-0.06	0.0499	6	6	12

eTable 2 – Robustness testing of differences in analytes between survivors and non-survivors in IPAH cohorts 1 and 2. To assess robustness of differences in analytes between survivors and non-survivors in these cohorts, 18 re-sampling analyses were performed, repeating the analysis each time removing 1/6 of patients in 3 randomised blocks, such that each sample was left out of 3 analyses. Proteins were then ranked by the number of times they met a p-value of <0.05 and those that were found significant in at least 33/36 analyses were selected for further study.

	Cohort 1			Cohort 2		
**Test Result Variable(s)**	**Area**	***SEM***	**Sig.**	**Area**	***SEM***	**Sig.**
**Higher value indicates mortality**						
BNP32	0.774	*0.063*	0.0002	0.787	*0.055*	1.9E-05
ST2	0.724	*0.067*	0.0022	0.806	*0.049*	5.2E-06
TIMP1	0.738	*0.059*	0.0011	0.792	*0.052*	1.4E-05
XEDAR	0.692	*0.063*	0.0086	0.809	*0.051*	4.0E-06
TIMP2	0.720	*0.062*	0.0026	0.766	*0.056*	7.6E-05
Epo	0.774	*0.061*	0.0002	0.666	*0.063*	0.0131
NRP1	0.736	*0.052*	0.0012	0.683	*0.063*	0.0063
FactorD	0.722	*0.055*	0.0023	0.695	*0.062*	0.0036
a1Antitrypsin	0.714	*0.071*	0.0034	0.700	*0.062*	0.0029
IGFBP1	0.697	*0.067*	0.0069	0.701	*0.061*	0.0028
**Lower value indicates mortality**						
Growth hormone receptor	0.736	*0.067*	0.0012	0.779	*0.055*	0.0000
Plasminogen	0.767	*0.058*	0.0003	0.752	*0.057*	0.0002
BMP1	0.754	*0.060*	0.0005	0.754	*0.056*	0.0002
Prekallikrein	0.800	*0.057*	3.9E-05	0.706	*0.061*	0.0022
RET	0.780	*0.061*	0.0001	0.727	*0.059*	0.0007
CNDP1	0.775	*0.061*	0.0002	0.722	*0.063*	0.0009
ApoE	0.773	*0.058*	0.0002	0.684	*0.062*	0.0061
Leptin	0.739	*0.061*	0.0011	0.689	*0.061*	0.0048
FactorH	0.754	*0.063*	0.0005	0.669	*0.063*	0.0120
ASAH2	0.685	*0.065*	0.0112	0.700	*0.061*	0.0029

eTable 3 – Performance of prognostic analytes by ROC analysis.

Proteins	Significance: Survivors vs non-survivors	
	Cohort 1	Cohort 2
BNP-32	0.000132	1.81E-05
IL-1 R4	0.003625	1.46E-06
TIMP-1	0.002162	5.16E-05
Growth hormone receptor	0.002795	5.56E-05
Plasminogen	0.005992	0.000662
BMP-1	0.009586	0.000685
Prekallikrein	0.001615	0.005796
RET	0.001488	0.000817
CNDP1	0.000428	0.001721
TIMP-2	0.002017	0.000101
Leptin	0.003521	0.003724
Factor D	0.005622	0.008352
Apo E	0.000742	0.004597
NRP1	0.002535	0.015836
a1-Antitrypsin	0.004298	0.005691
Epo	0.000236	0.026144
IGFBP-1	0.006922	0.003441
XEDAR	0.041376	9.73E-06
Factor H	0.002662	0.008311
ASAH2	0.0073	0.007768

eTable 4 - Sensitivity analysis excluding HPAH patients. Discovery and validation comparisons of protein levels in survivors and non-survivors were performed again excluding 7 HPAH cases. Significance of top 20 robustly prognostic proteins (identified by analysis presented in eTable 2) are shown.

Analyte	Percentile of cut-off in discovery IPAH cohorts 1+2	Equivalent concentration from lab assays in validation IPAH cohort 4	
Reduction in protein indicates increased risk			
Apo E	0.454	38.13	ug/ml
Factor H	0.463	263.7	ug/ml
Plasminogen	0.514	420.9	ug/ml
Increase in protein indicates increased risk			
Epo	0.674	31.91	mIU/ml
Factor D	0.537	1733	ng/ml
IGFBP-1	0.697	26.94	ng/ml
ST2	0.807	43.09	ng/ml
TIMP-1	0.312	138.9	ng/ml
TIMP-2	0.638	357.9	ng/ml

eTable 5 – ROC-derived cut-offs for each of the 9 independent, prognostic proteins validated by alternative assays. Concentrations were derived from percentile of ROC-derived cut-off in SomaScan data, i.e. if the optimal cut-off in the SomaScan data indicated 60% of patients with highest levels of the marker were at risk, the value identifying the top 60% of patients determined by the equivalent lab assay is given.

	Change in Panel score		
	**No increase**	**Increase**	
	**Median (IQR) or frequencies**		**Sig.**
Age at diagnosis	43.2 (30.0 - 57.0)	52.3 (29.3 - 63.2)	0.43
Mean pulmonary artery pressure, mmHg	53.3 (45.3 - 59.8)	47 (41 - 51)	0.74
Pulmonary vascular resistance, dynes/cm5/min	1071.6 (781.8 - 1216.8)	678.2 (524.8 - 921.2)	0.17
Venous oxygen saturations, %	60 (56.5 - 63.8)	62.9 (52.5 - 68.3)	0.29
Mean right atrial pressure, mmHg	7.5 (3 - 11)	6 (4 - 7)	0.66
Cardiac index, L/min/kg/m2	2.17 (1.6 - 2.6)	2.17 (2.0 - 2.6)	0.24
Pulmonary artery wedge pressure, mmHg	7 (4 - 9)	9 (6 - 10)	0.43
NYHA Functional Class, II / III / IV	5 / 17 / 6	1 / 11 / 3	0.83
Single/combination therapy	25/3	15/0	0.19

eTable 6 - Clinical characteristics of patients whose protein panel scores did or did not increase after initiation of therapy.

	Hazard ratio	95% CI	Sig.
**Development (Cohorts 1 + 2)**			
Panel of 9 proteins	2.64	1.94 - 3.58	6E-10
NT-proBNP	1.49	1.16 - 1.91	0.002
**Validation (Cohort 4)**			
Panel of 9 proteins	1.94	1.27 - 2.98	0.002
NT-proBNP	1.37	0.95 - 1.98	0.096

eTable 7 - Cox regression models of panel score against established prognostic marker, NT-proBNP.

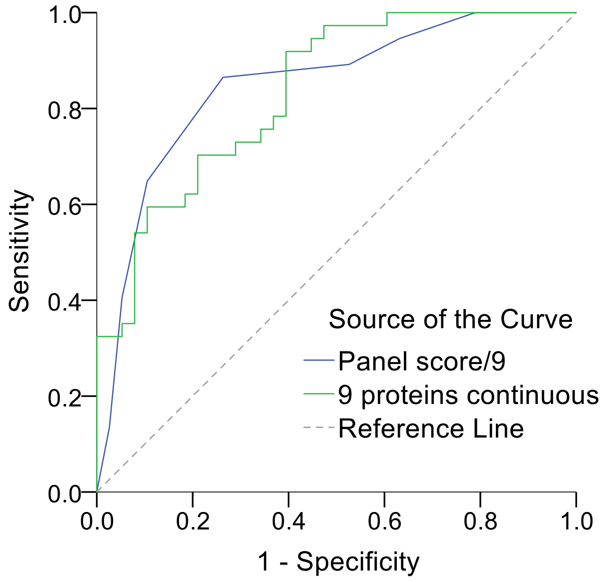

Test Result Variable(s)	Area	Sig.	95% CI	
Panel score/9	0.85	2.13E-07	0.76	0.94
9 proteins continuous	0.83	6.34E-07	0.75	0.92

eFigure 1 - ROC analysis of 2.5 year survival in cohort 2 predicted by the 9 prognostic proteins, either as a simplified score out of 9 based on cut-offs or using an equation which uses the continuous measurements of each protein for each patient.

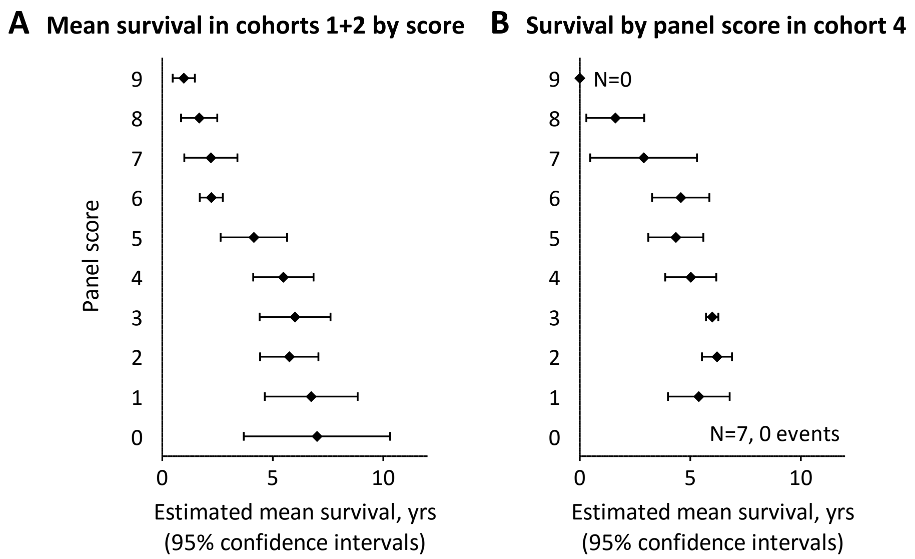

eFigure 2 – Mean survival estimates in patients from A. discovery (cohorts 1 and 2) and B. validation (cohort 4) divided by panel score.

Prognostic performance of the panel score after removing single variables

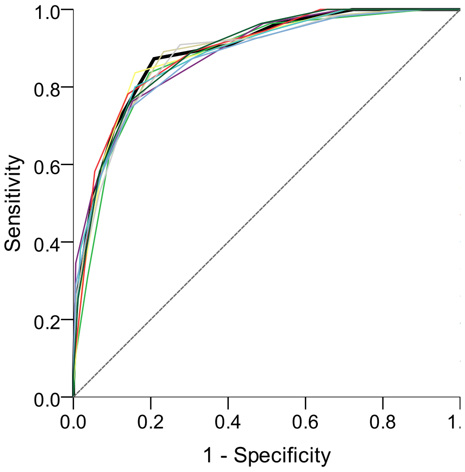

Variable removed	AUC	Sig.	95% CI Lower	95% CI Upper
None	0.89	5.30E-18	0.842	0.938
Factor D	0.89	6.87E-18	0.841	0.937
Factor H	0.89	8.34E-18	0.841	0.934
TIMP1	0.89	1.00E-17	0.837	0.936
Epo	0.89	1.43E-17	0.836	0.934
TIMP2	0.88	1.69E-17	0.835	0.933
IGFBP1	0.88	2.05E-17	0.833	0.933
Plasminogen	0.88	4.44E-17	0.827	0.931
ApoE	0.87	1.30E-16	0.819	0.927
IL-1 R4	0.87	1.33E-16	0.821	0.925

Prognostic performance of the panel score after removing two variables

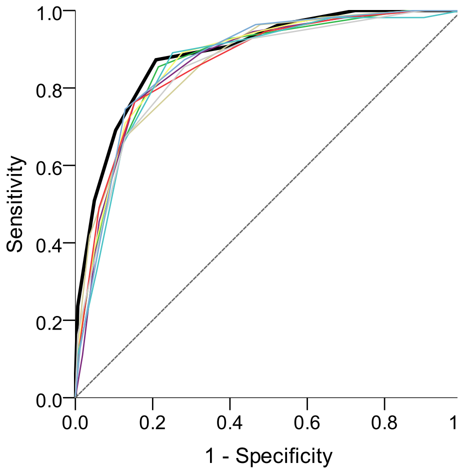

Variables removed	AUC	Sig.	95% CI Lower	95% CI Upper
None	0.89	5.30E-18	0.842	0.938
IL1-R4 + Factor D	0.87	1.60E-16	0.821	0.923
IL1-R4 + Factor H	0.87	1.76E-16	0.821	0.922
IL1-R4 + TIMP1	0.87	3.50E-16	0.815	0.921
IL1-R4 + Epo	0.87	6.38E-16	0.811	0.918
IL1-R4 + TIMP2	0.86	7.50E-16	0.811	0.917
IL1-R4 + IGFBP1	0.86	8.05E-16	0.809	0.918
IL1-R4 + Plasminogen	0.86	1.66E-15	0.803	0.915
IL1-R4 + ApoE	0.85	5.29E-15	0.796	0.91

eFigure 3 – ROC analysis of panel score following removal of any 1 or 2 proteins from the scoring.

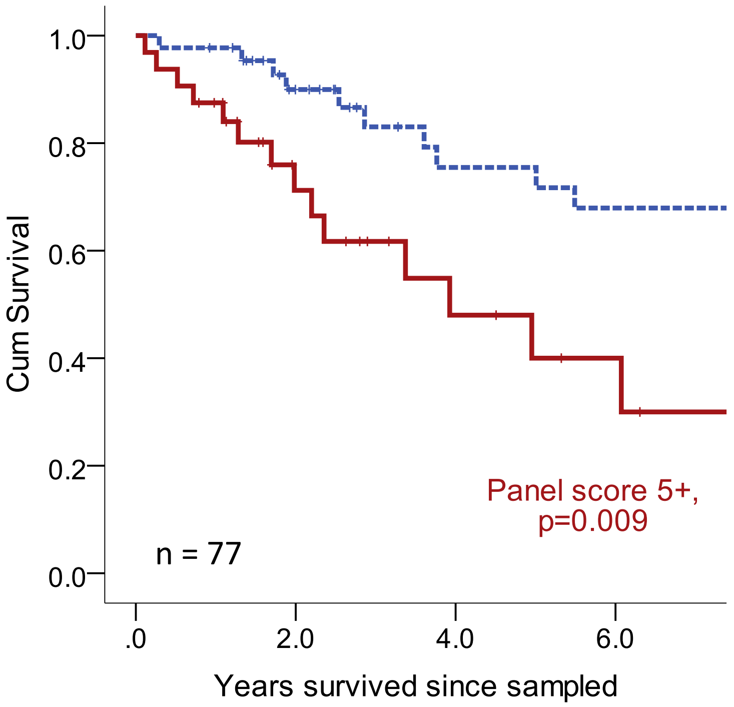

eFigure 4 – Sub-analysis of protein panel in patients naïve to PAH targeted therapies. 40 additional samples from patients in Cohort 1 before they commenced therapy were analysed in addition to the patients already analysed before therapy. Kaplan-Meier analysis shows estimated survival over time in treatment-naïve IPAH patients divided by panel score.

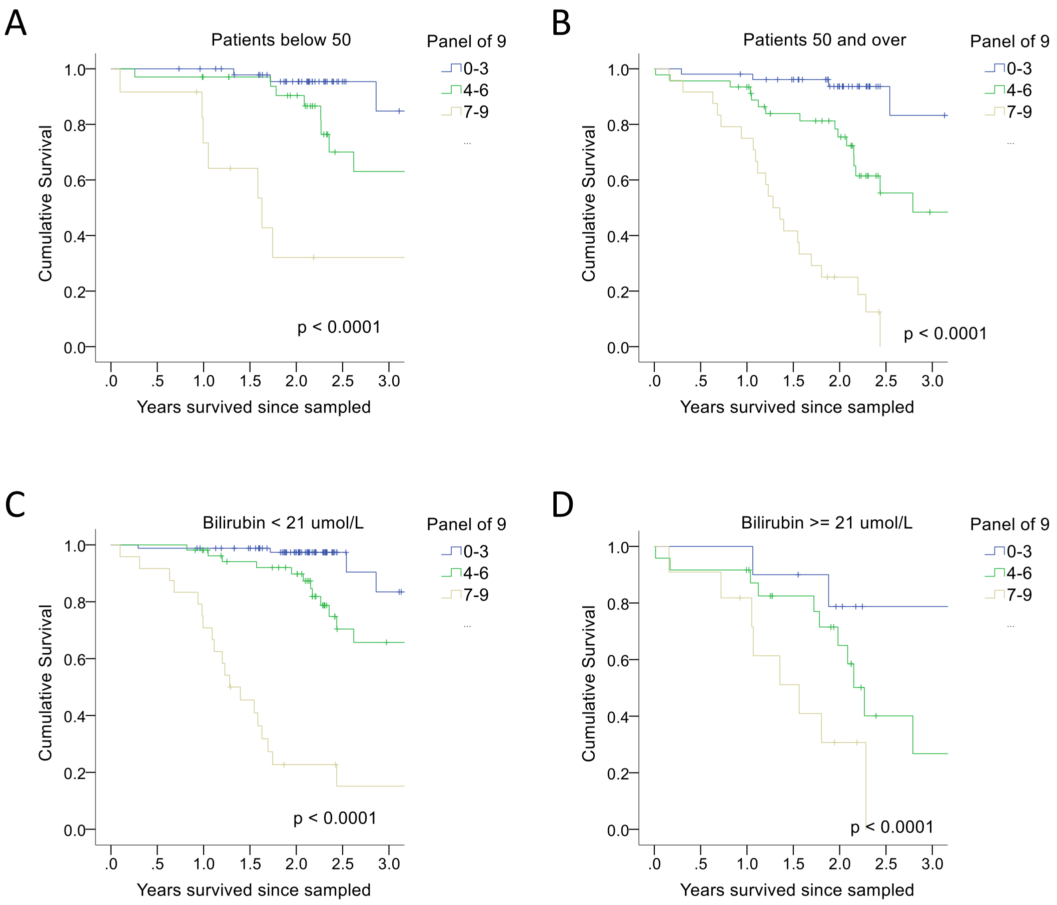

eFigure 5 - Survival by panel score in PAH patients from cohorts 1 and 2 divided by age and bilirubin levels. A. Patients below 50. B. Patients 50 and above. C. Patients with bilirubin levels below 21 µmol/L and D. above 21 µmol/L.

Prognostic performance of changes in variables measured at diagnostic and follow-up catheterisations

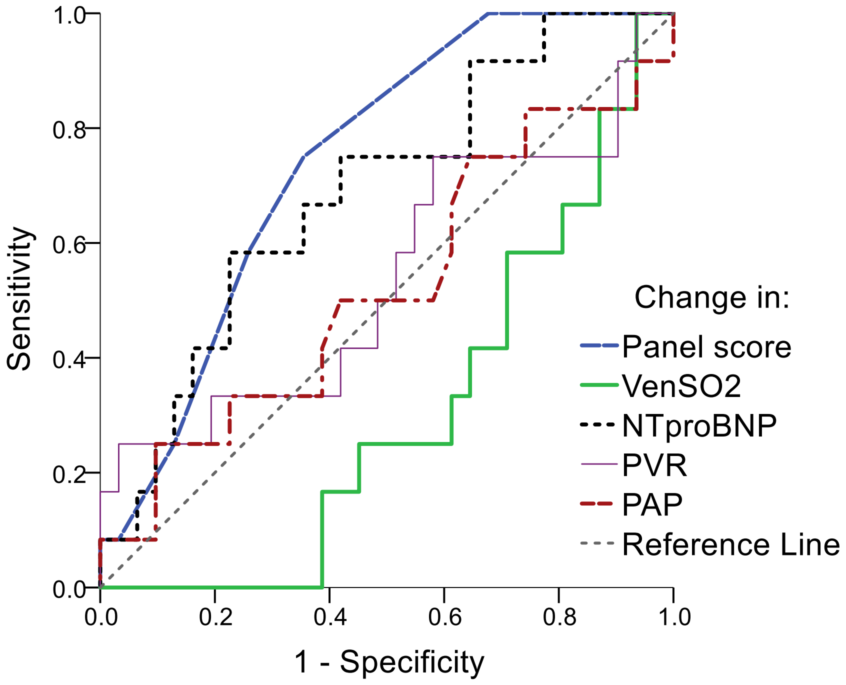

Change in:	AUC	Sig.	95% CI
Panel score	0.742	0.015	0.59 - 0.89
VenSO2	0.306	0.051	0.15 - 0.47
NTproBNP pmol/L	0.688	0.058	0.52 - 0.86
PVR	0.54	0.685	0.33 - 0.75
PAP	0.523	0.818	0.32 - 0.73

eFigure 6 – ROC analysis of change in panel score, venous oxygen saturations (VenSO2), NT-proBNP, pulmonary vascular resistance (PVR) and mean pulmonary artery pressure (PAP) from diagnostic catheterisation to follow-up after initiation of targeted therapies.

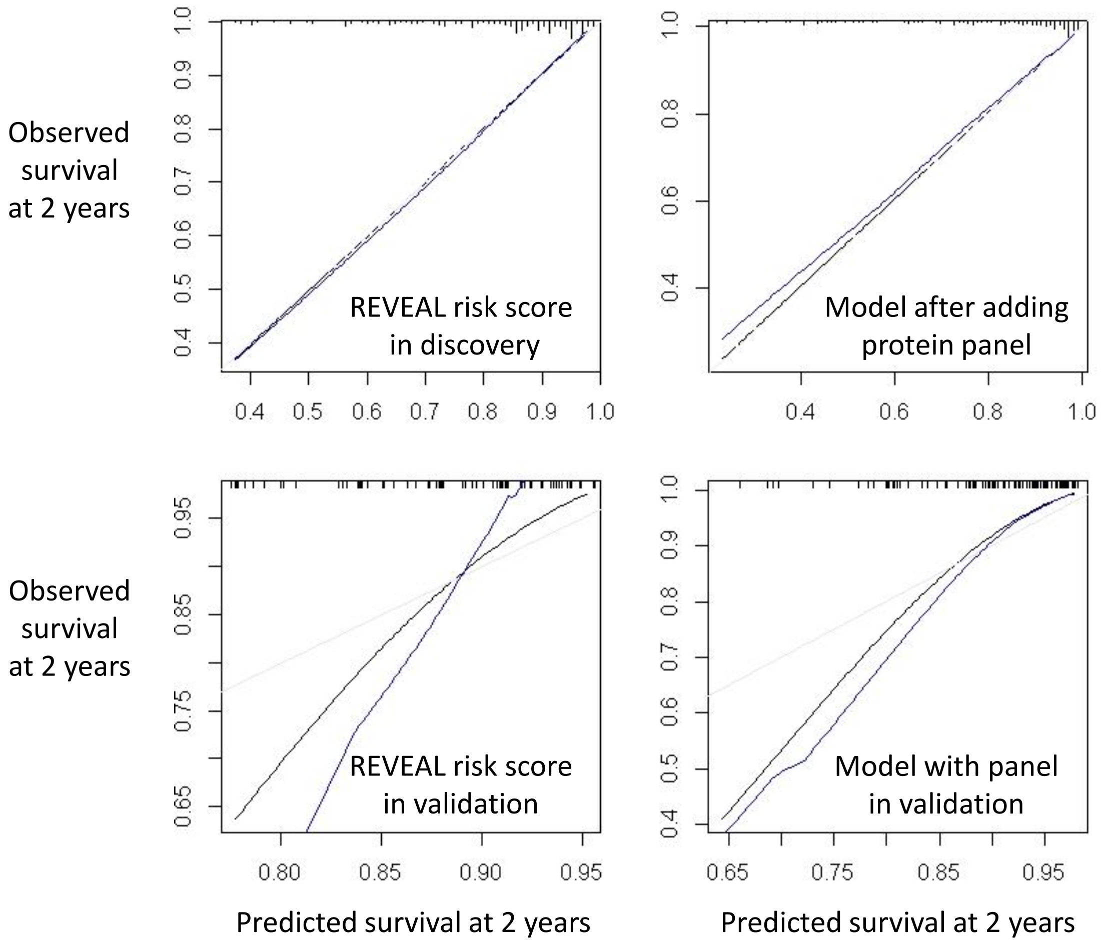

eFigure 7 - Calibration plots for Cox models. Each plot indicates the calibration between predicted and expected mortality at 2 years before (black) and after (blue) correcting for optimism. The grey line in each plot indicates the ideal of observed=predicted. The dashes at the top of each plot indicate predicted mortality for individuals included in the study. The validation plots are slightly skewed at lower predicted risks where there were few patients.

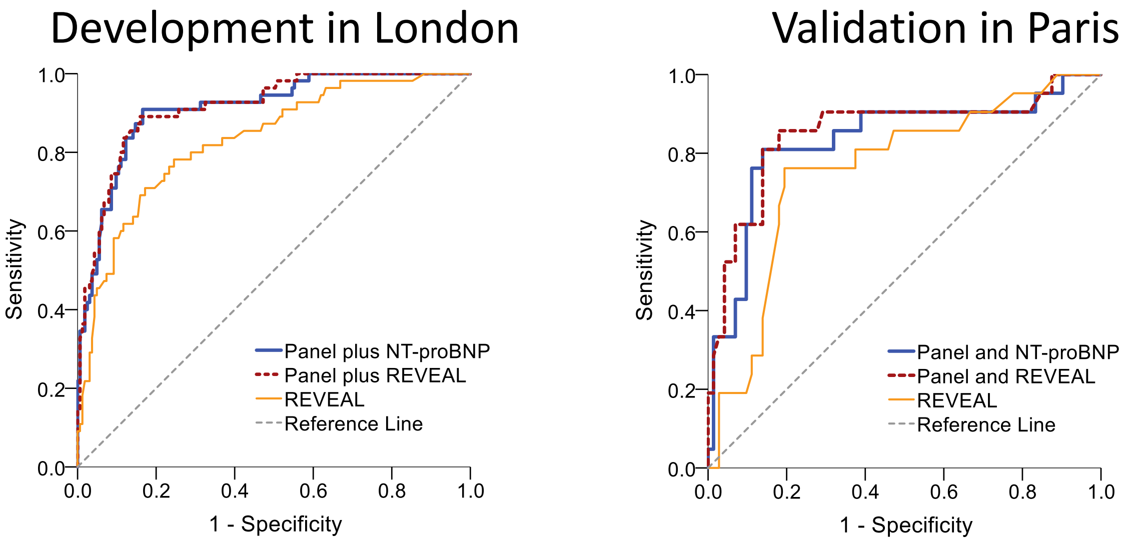

	AUC	95% CI	Sig.
Panel and REVEAL	0.91	0.87 - 0.96	4E-20
Panel and NT-proBNP	0.91	0.87 - 0.95	8E-20
REVEAL	0.83	0.77 - 0.89	2E-13

	AUC	95% CI	Sig.
Panel and REVEAL	0.85	0.74 - 0.96	9E-07
Panel and NT-proBNP	0.83	0.72 - 0.95	4E-06
REVEAL	0.76	0.64 - 0.88	3E-04

eFigure 8 - ROC analysis of panel score added to REVEAL equation or NT-proBNP compared to REVEAL equation alone.
